# Patterns of leisure-time physical activity across pregnancy and adverse pregnancy outcomes

**DOI:** 10.1186/s12966-018-0701-5

**Published:** 2018-07-11

**Authors:** Janet M. Catov, Corette B. Parker, Bethany Barone Gibbs, Carla M. Bann, Benjamin Carper, Robert M. Silver, Hyagriv N. Simhan, Samuel Parry, Judith H. Chung, David M. Haas, Ronald J. Wapner, George R. Saade, Brian M. Mercer, C. Noel Bairey-Merz, Philip Greenland, Deborah B. Ehrenthal, Shannon E. Barnes, Anthony L. Shanks, Uma M. Reddy, William A. Grobman, for the NICHD NuMoM2b and NHLBI NuMoM2b Heart Health Study Network

**Affiliations:** 1 Department of Obstetrics, Gynecology and Reproductive Sciences, University of Pittsburgh School of Medicine, 204 Craft Avenue, Suite A208, Pittsburgh, PA 15213 USA; 2 RTI International, Research Triangle Park, NC USA; 3 Department of Health and Physical Activity, University of Pittsburgh, Pittsburgh, PA USA; 4 Department of Obstetrics and Gynecology, University of Utah and Intermountain Healthcare, Salt Lake City, UT USA; 5 Department of Obstetrics and Gynecology, University of Pennsylvania, Philadelphia, PA USA; 6 Department of Obstetrics and Gynecology, University of California, Irvine, Irvine, CA USA; 7 Department of Obstetrics and Gynecology, School of Medicine, Indiana University, Indianapolis, IN USA; 8 Department of Obstetrics and Gynecology, Columbia University, New York, NY USA; 9 Department of Obstetrics and Gynecology, University of Texas Medical Branch, University of Texas, Galveston, TX USA; 10 Department of Obstetrics and Gynecology, Case Western Reserve University, Cleveland, OH USA; 11 Division of Cardiology, Department of Medicine, Cedars-Sinai Medical Center, Los Angeles, CA USA; 12 Division of Cardiology, Department of Medicine, Northwestern University, Chicago, IL USA; 13 Department of Medicine, University of Wisconsin-Madison, Madison, WI USA; 14 Eunice Kennedy Shriver National Institute of Child Health and Human Development, Bethesda, MD USA; 15 Department of Obstetrics, Gynecology-Maternal Fetal Medicine & Preventive Medicine, Northwestern University, Chicago, IL USA

**Keywords:** Pregnancy, Physical activity, Gestational diabetes, Preterm birth

## Abstract

**Background:**

Although leisure-time physical activity (PA) contributes to overall health, including pregnancy health, patterns across pregnancy have not been related to birth outcomes. We hypothesized that women with sustained low leisure-time PA would have excess risk of adverse pregnancy outcomes, and that changing patterns across pregnancy (high to low and low to high) may also be related to risk of adverse pregnancy outcomes.

**Methods:**

Nulliparous women (*n* = 10,038) were enrolled at 8 centers early in pregnancy (mean gestational age in weeks [SD] = 12.05 [1.51]. Frequency, duration, and intensity (metabolic equivalents) of up to three leisure activities reported in the first, second and third trimesters were analyzed. Growth mixture modeling was used to identify leisure-time PA patterns across pregnancy. Adverse pregnancy outcomes (preterm birth, [PTB, overall and spontaneous], hypertensive disorders of pregnancy [HDP], gestational diabetes [GDM] and small-for-gestational-age births [SGA]) were assessed via chart abstraction.

**Results:**

Five patterns of leisure-time PA across pregnancy were identified: High (35%), low (18%), late decreasing (24%), early decreasing (10%), and early increasing (13%). Women with sustained low leisure-time PA were younger and more likely to be black or Hispanic, obese, or to have smoked prior to pregnancy. Women with low vs. high leisure-time PA patterns had higher rates of PTB (10.4 vs. 7.5), HDP (13.9 vs. 11.4), and GDM (5.7 vs. 3.1, all *p* < 0.05). After adjusting for maternal factors (age, race/ethnicity, BMI and smoking), the risk of GDM (Odds ratio 2.00 [95% CI 1.47, 2.73]) remained higher in women with low compared to high patterns. Early and late decreasing leisure-time PA patterns were also associated with higher rates of GDM. In contrast, women with early increasing patterns had rates of GDM similar to the group with high leisure-time PA (3.8% vs. 3.1%, adjusted OR 1.16 [0.81, 1.68]). Adjusted risk of overall PTB (1.31 [1.05, 1.63]) was higher in the low pattern group, but spontaneous PTB, HDP and SGA were not associated with leisure-time PA patterns.

**Conclusions:**

Sustained low leisure-time PA across pregnancy is associated with excess risk of GDM and overall PTB compared to high patterns in nulliparous women. Women with increased leisure-time PA early in pregnancy had low rates of GDM that were similar to women with high patterns, raising the possibility that early pregnancy increases in activity may be associated with improved pregnancy health.

**Trial registration:**

Registration number NCT02231398.

**Electronic supplementary material:**

The online version of this article (10.1186/s12966-018-0701-5) contains supplementary material, which is available to authorized users.

## Background

Physical activity contributes to overall health, including pregnancy health [1]. Similar to non-pregnant adults, the American College of Obstetricians and Gynecologists recommends that pregnant women achieve 20 to 30 min of moderate intensity activity per day on most or all days of the week [2]. Pregnancy, however, is also a time of increased sedentary behavior due to physical, individual and clinical determinants [3, 4]. Individual patterns of activity across pregnancy have not been well described. Most data across gestation are derived from cross sectional assessments in different women that are aggregated to describe behaviors in each trimester [4, 5].

Preterm birth (delivery < 37 weeks gestation), hypertensive disorders of pregnancy (elevated blood pressure with or without proteinuria), gestational diabetes (de novo abnormal glucose metabolism) and fetal growth restriction (birth weight < 5th percentile) complicate up to 38% of first births [6] and are associated with significant maternal and neonatal morbidity and mortality. These adverse pregnancy outcomes are heterogeneous conditions, with some overlapping vascular and metabolic pathophysiologies including endothelial dysfunction, insulin resistance, and inflammation [7–10]. Physical activity affects each of these factors, which may be mediating links between increased physical activity and reduced risk of complications [11, 12]. Importantly, the risk of adverse outcomes is highest in first pregnancies, and a history of prior complications is the strongest determinant of recurrence in subsequent births [13, 14]. Thus, health behaviors in a first pregnancy are a unique opportunity to improve immediate and long-term maternal and child health.

In a prospective cohort of 10,038 nulliparous women enrolled at 8 centers in the U.S., we set out to describe patterns of self-reported leisure-time physical activity (PA) and related these patterns to occurrence of preterm birth, hypertensive disorders of pregnancy, gestational diabetes and small-for-gestational-age births. We hypothesized that women with sustained levels of low leisure-time PA would have higher risk of adverse pregnancy outcomes. We also considered that changing patterns across pregnancy may also be important, such that increases in activity may be beneficial and decreases may be associated with higher risk of adverse outcomes.

## Methods

The Nulliparous Pregnancy Outcomes Study: Monitoring Mothers-to-Be (nuMoM2b) is a prospective cohort study designed to identify factors that contribute to adverse pregnancy outcomes [15]. This prospective cohort study enrolled 10,038 nulliparous women with singleton pregnancies from 8 clinical centers in the United States (Case Western Reserve University; Columbia University; Indiana University; University of Pittsburgh; Northwestern University; University of California at Irvine; University of Pennsylvania; and University of Utah). In brief, women were eligible for enrollment if they had a viable singleton gestation, had no previous pregnancy that lasted more than 20 weeks of gestation, and were between 6 0/7 weeks of gestation and 13 6/7 weeks of gestation at recruitment. Exclusion criteria were maternal age younger than 13 years, history of three or more spontaneous abortions, current pregnancy complicated by a suspected fatal fetal malformation, known fetal aneuploidy, assisted reproduction with a donor oocyte, multifetal reduction, or plans to terminate the pregnancy. Nulliparity was defined as having had no prior pregnancy lasting 20 weeks or more based on self-report. All local institutional review boards approved the study protocol, and participants provided written informed consent prior to enrollment.

Leisure-time PA during pregnancy was self-reported at a study visit in each trimester (6- < 14, 16- < 22, and 22- < 30 weeks gestation, with the third visit occurring at least 4 weeks after the previous visit) using standardized physical activity questions adapted from the Behavior Risk Factor Surveillance System (BRFSS) [16, 17]. Women were asked whether they participated in any leisure-time PA during the previous four weeks. If yes, they were asked to describe the activity in which they spent the most time and to provide information on the number of times per week they had taken part in this activity over the four weeks, and how many minutes per time. For running, jogging, walking, cycling and swimming, they were also asked about distance. This was repeated for the second and third activity in which they spent the most time. By design, these questions assess structured, physical activities which have been linked to health, including pregnancy health [18, 19]. Each activity was assigned an intensity level [metabolic equivalent (MET)] by trained coders based on the Physical Activity Compendium [20]. This MET value was multiplied by the frequency and duration of each activity to obtain volume for each activity (MET-minutes per week), and then summed across all activities reported by the participant. In addition to analysis of MET-minutes per week, adequacy of a participant’s exercise regimen was assessed as ≥150 min of moderate activity per week, ≥75 min of vigorous activity per week, or an equivalent combination of the two. Moderate activity was defined as leisure-time PA with an intensity 3 ≤ METs< 6 and vigorous activity was defined as METs≥6. A total of 10,022 women provided activity data at one or more visits; 10,016 at Visit 1; 9408 at Visit 2; and 9215 at Visit 3. For all 3 visits, the median (Q1, Q3) was 1 (0,2) for number of activities reported.

Adverse pregnancy outcomes were adjudicated from medical record abstraction, performed by certified research personnel. Quality control checks via re-abstraction were performed by the site principal investigator on a random selection of charts with and without complications. For 82% of the charts reviewed, no discrepancies were found. For those charts with discrepancies between the two abstractions, these differences were generally minor and not related to the primary adverse pregnancy outcomes. Preterm births were those delivered prior to 37 weeks, and further classified as spontaneous if after spontaneous onset of labor or premature rupture of membranes. Hypertensive disease of pregnancy included preeclampsia with and without severe features, super-imposed preeclampsia, eclampsia, and gestational hypertension, as defined according to established criteria [21]. Gestational diabetes mellitus was defined by one of the following glucose tolerance testing (GTT) criteria: fasting 3-h 100 g GTT with two abnormal values [fasting 95 mg/dL or greater, 1-h 180 mg/dL or greater, 2-h 155 mg/dL or greater, 3-h 140 mg/dL or greater]; 2) fasting 2-h 75 g GTT with one abnormal value [fasting 92 mg/dL or greater, 1-h 180 mg/dL or greater, 2-h 153 mg/dL or greater]; or 3) nonfasting 50-g GTT 200 mg/dL or greater if no fasting 3-h or 2-h GTT was performed [22]. If no GTT data were available, the clinical diagnosis from chart abstraction was used for GDM classification. Small-for-gestational-age (SGA) birthweight was defined as <5th percentile for gestational age at delivery based on Alexander fetal growth curves [23]. Analysis was restricted to pregnancies carried 20 or more weeks of gestation. Women with pre-existing diabetes were identified via chart abstraction of the medical record from the delivery hospitalization (*n* = 151) and were excluded from analysis of gestational diabetes.

Covariates were recorded at the enrollment study visit and included maternal age, race/ethnicity, nausea, vomiting, retching in the 12 h prior to the study visit interview (as derived from the PUQE survey), [24] smoking during the 3 months prior to pregnancy, chronic hypertension or pre-existing diabetes, and education. Early pregnancy body mass index (BMI, kg/m^2^) was based on measured weight and height, which is highly correlated with pre-pregnancy BMI.

Patterns of leisure-time PA throughout pregnancy were identified using growth mixture modeling [25] conducted in Mplus version 8 with missing values addressed using full information maximum likelihood [26]. Models included leisure-time PA values (METs-minutes per week) from the three study visits. Due to the skewness of the distributions of total weekly MET-minutes, we applied a log transformation. Having determined the most appropriate form of the growth curve (linear vs. quadratic), we compared the fit of growth mixture models with one- to five-class solutions. Model fit was assessed based on standard fit indices (i.e., AIC, BIC, and sample-adjusted BIC); the inclusion of additional classes was evaluated using likelihood ratio tests. In addition, the number of participants in each class was examined, requiring a minimum of 5–10% of participants per class to avoid the inclusion of spurious classes. Final model selection was informed by the statistical results and interpretation of the classes. Specifically, we determined that a 5-group solution most ideally fit the data based on the statistical results and the fact that patterns matched what is generally known about activity across gestation. For example, this 5-group solution included a group with zero or very low leisure-time PA across pregnancy, while other groups, on average, reported reductions as pregnancy advanced.

We summarized maternal characteristics according to leisure-time PA pattern group, and compared these characteristics using chi square tests and analysis of variance F-tests. We compared occurrence of adverse outcomes in each leisure-time PA pattern group using logistic regression, and adjusted differences for age (linear and quadratic terms), race/ethnicity, early pregnancy BMI (linear and quadratic terms), and smoking status 3 months prior to pregnancy which were covariates selected a priori. As sensitivity analyses, we conducted additional adjusted analyses adding the probability of pattern group membership taken from the growth mixture model to the covariates in the logistic regression model. Odds ratios from all logistic regression models were computed with the most active class as the referent group.

## Results

**Fig. 1:**
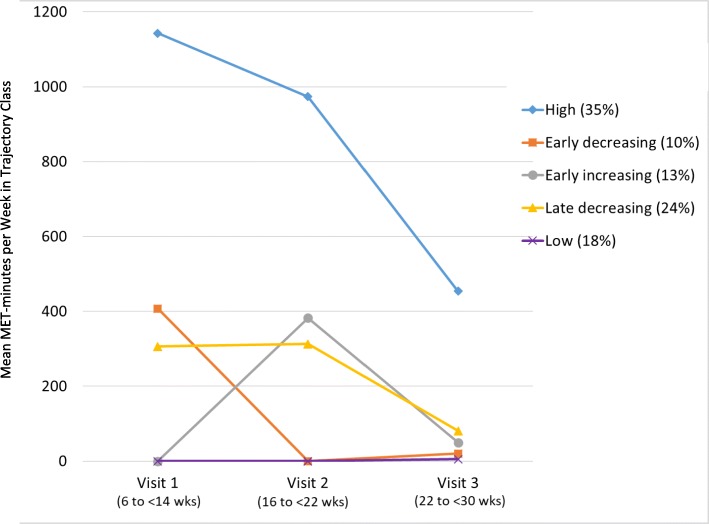
Leisure-time physical activity per week for nuMoM2b study visits

Growth mixture modeling revealed five patterns of self-reported leisure-time PA across pregnancy (Fig. 1). The largest group of women reported high levels of leisure-time PA in the first trimester that decreased to moderate levels in the third trimester (high, 35%). One group reported no leisure-time PA during any trimester of pregnancy (low, 18%). Another group reported moderate levels in the first and second trimester that decreased to low levels in the third trimester (late decreasing, 24%). Some women increased leisure-time PA in the first half of pregnancy, then decreased to low levels (early increasing, 13%) while another group decreased leisure-time PA in the first half of pregnancy and reported persistently low levels in the third trimester (early decreasing, 10%). Of note, had we evaluated leisure-time PA in a cross-sectional fashion at each trimester, different groups would have emerged. For example, in the first trimester women clustered in three groups (high, moderate, low); in the second trimester, there were also three cross sectional groups but comprised of different women.

The most common activities reported for each group were walking, aerobics, yoga, weight lifting and running (Table 1). We summarized the average minutes of leisure-time PA per week in each group across gestation to translate the patterns detected into clinically meaningful metrics (Table 2). Almost one quarter of women with high leisure-time PA achieved recommended levels of activity early in pregnancy (≥150 min/weeks of moderate activity per week, or ≥ 75 min/week of vigorous activity, or an equivalent combination of the two) [2]. The overall patterns of increasing or decreasing leisure-time PA were similar when summarized according to guidelines, and generally, fewer than 5% of women in these groups achieved recommended levels at any assessment.

**Table 1:** Counts of most frequent activities reported by nuMoM2b study visit^a^

Activity (METs)	Visit 1	Visit 2	Visit 3	Across visits
	6 to < 14 wks (N = 10,016)	16 to < 22 wks (*N* = 9408)	22 to < 30 wks (*N* = 9215)	
Walking (3.5)^b^	5364	5554	5390	16,308
Aerobics class/Exercise machines (5.5)	1393	1255	1107	3755
Yoga (3.0)	825	1098	1107	3030
Weight lifting (3.5)	651	624	497	1772
Running(8.0)^b^	697	481	215	1393
Swimming (7.0)	383	350	390	1123
Cycling (7.5)^b^	400	314	197	911
Calisthenics/Home or Gym Exercise (4.0)	277	264	212	753
Hiking/Backpacking (6.5)	154	132	102	388
Dancing (5.0)	148	125	77	350
Gardening (3.8)	117	76	61	254
Stair climbing (6.4)	48	58	52	158
Tennis (7.3)	37	26	11	74
Volleyball (4.0)	42	21	11	74
Judo/Karate/Tae Kwon Do (7.8)	26	21	22	69
Softball/baseball (5.0)	33	22	6	61
Basketball (6.5)	30	15	11	56
Sledding/Tobogganing (7.0)	29	24	2	55
Soccer/Badminton/Racquetball (7.0)	33	12	4	49

^a^Study visits occurred in time according to the gestational age of participants

^b^For walking, jogging, running, and cycling, METs were based on speed when distance and duration were provided. Assigned values are shown below

**Walking**

no speed (general) = 3.5 METs

< 2.0 mph = 2.0 METs

2.0–2.4 mph = 2.8 METs

2.5–2.7 mph = 3.0 METs

2.8–3.5 mph = 3.5 METs

3.6–4.2 mph = 5.0 METs

4.3–4.9 mph = 7.0 METs

≥ 5.0 mph = 8.3 METs

**Jogging/Running**

jogging no speed (general) = 7.0 METs

running no speed (general) = 8.0 METs

< 4.9 mph = 6.0 METs

5.0–5.9 mph = 9.0 METs

6.0–6.9 mph = 11 .0 METs

7.0–7.9 mph = 11.5 METs

8.0–8.9 mph = 12.0 METs

9.0–9.9 mph = 12.8 METs

≥ 10 mph = 15.0 METs

**Cycling**

no speed (general) = 7.5 METs

< 6.0 mph = 3.5 METs

6.0 - 9.9 mph = 5.0 METs

10.0 - 11.9 mph = 6.8 METs

12.0 - 13.9 mph = 8.0 METs

14.0 - 15.9 mph = 10.0 METs

16.0 - 19.9 mph= 12.0 METs

≥ 20.0 mph = 15.8 METs

**Table 2:** Number and percent of women with adequate exercise regimen^a^ within nuMoM2b study visit by leisure-time physical activity trajectory class across study visits

Leisure-time physical activity trajectory class	Visit 1	Visit 2	Visit 3
	6 to < 14 wks	16 to < 22 wks	22 to < 30 wks
	(*N* = 10,016)	(*N* = 9408)	(*N* = 9215)
High	833/3479 (23.9%)	671/3274 (20.5%)	470/3231 (14.5%)
Early decreasing	55/1017 (5.4%)	2/1009 (0.2%)	22/981 (2.2%)
Early increasing	9/1338 (0.7%)	66/1312 (5.0%)	50/1284 (3.9%)
Late decreasing	39/2402 (1.6%)	46/2249 (2.0%)	64/2210 (2.9%)
Low	5/1780 (0.3%)	5/1564 (0.3%)	12/1509 (0.8%)
*p*-value^2/^	<.0001	<.0001	<.0001

^a^Adequate exercise regimen is defined as > = 150 min of moderate activity per week or > =75 min of vigorous activity per week or some combination of moderate and vigorous that is equivalent to these guidelines. Moderate activity is 3 to < 6 METs. Vigorous activity is 6+ METs

^2/^*P*-values are shown for chi-square tests of association between leisure-time physical activity trajectory class and percent of women with adequate exercise regimen for the specific study visit

Women with high leisure-time PA, on average, were older, less likely to be of minority race/ethnicity, less likely to be obese, and reported less nausea, lower smoking rates and fewer chronic conditions prior to pregnancy compared to women in all other groups (Table 3). Women with late decreasing patterns were similar to this group in terms of race/ethnicity and smoking, but were more likely to be obese with higher rates of nausea and pre-pregnancy chronic conditions. Women with low leisure-time PA across pregnancy were younger, more likely to be of minority race/ethnicity, and had higher rates of smoking compared to other groups. Those with early pregnancy changes in leisure-time PA (early increasing, early decreasing) were similar to each other, with about half of the group of minority race/ethnicity, a third obese, and a majority with early pregnancy nausea and smoking. However, women with early increasing patterns were less likely to be overweight compared to their counterparts with early decreasing patterns.

**Table 3:** Baseline characteristics by leisure-time physical activity trajectory class across nuMoM2b study visits

Baseline characteristics	Leisure-time physical activity trajectory class					*p*-value^1/^
	High	Early decreasing	Early increasing	Late decreasing	Low	
	(*N* = 3483)	(*N* = 1017)	(*N* = 1338)	(*N* = 2404)	(*N* = 1780)	
Maternal age, in years						
Mean (standard deviation)	28.8 (5.4)	25.5 (5.5)	25.8 (5.8)	26.9 (5.4)	25.2 (5.6)	<.0001
Category: *n* (%)						<.0001
13–21	427 (12.3)	301 (29.6)	372 (27.8)	478 (19.9)	551 (31.0)	
22–35	2735 (78.6)	682 (67.1)	886 (66.2)	1783 (74.2)	1131 (63.5)	
> 35	318 (9.1)	34 (3.3)	80 (6.0)	143 (5.9)	98 (5.5)	
Maternal race: *n* (%)						<.0001
White Non-Hispanic	2491 (71.6)	536 (52.7)	682 (51.0)	1540 (64.1)	740 (41.6)	
Black Non-Hispanic	290 (8.3)	180 (17.7)	256 (19.1)	330 (13.7)	362 (20.3)	
Hispanic	374 (10.7)	200 (19.7)	258 (19.3)	325 (13.5)	534 (30.0)	
Asian	151 (4.3)	35 (3.4)	62 (4.6)	95 (4.0)	64 (3.6)	
Other	174 (5.0)	66 (6.5)	80 (6.0)	114 (4.7)	80 (4.5)	
Early Pregnancy BMI, in kg/m^2^						
Mean (standard deviation)	25.2 (5.3)	27.4 (6.9)	27.0 (6.8)	26.9 (6.8)	26.8 (6.4)	<.0001
Category: *n* (%)						<.0001
< 25	2112 (61.7)	442 (44.2)	663 (50.3)	1180 (49.9)	797 (46.8)	
25 to < 30	803 (23.4)	282 (28.2)	293 (22.2)	586 (24.8)	480 (28.2)	
≥ 30	510 (14.9)	275 (27.5)	361 (27.4)	601 (25.4)	426 (25.0)	
Nausea, vomiting, retching in 12 h prior V1 interview: *n* (%)	1704 (49.0)	592 (58.2)	789 (59.0)	1317 (54.8)	1016 (57.1)	<.0001
Smoked during 3 months prior to pregnancy: *n* (%)	487 (14.0)	223 (21.9)	288 (21.6)	392 (16.3)	392 (22.1)	<.0001
Chronic hypertension: *n* (%)	43 (1.3)	36 (3.7)	36 (2.8)	84 (3.7)	43 (2.6)	<.0001
Pre-gestational diabetes: *n* (%)	30 (0.9)	25 (2.5)	29 (2.2)	51 (2.2)	16 (1.0)	<.0001

^1/^*p*-values are shown for chi-square tests (percent) and from ANOVA F-tests (mean [SD])

Rates of adverse outcomes differed across groups (Table 4). The group of women with low leisure-time PA had higher rates of preterm birth, hypertensive disorders of pregnancy, gestational diabetes, and small for gestational age births compared to those with high activity patterns. Conversely, women with high leisure-time PA had the lowest rates of all complications. After accounting for maternal characteristics (age, race/ethnicity, BMI, smoking), rates of GDM differed according to these patterns. Those with low leisure-time PA (aOR 2.00 [95% CI 1.47, 2.73]), late decreasing (aOR 1.50 [1.12, 2.01]), and early decreasing patterns (aOR 1.74 [1.21, 2.52]) all had elevated risk of GDM. Of note, the group with early increasing leisure-time PA had rates of GDM similar to those with high activity patterns across pregnancy (3.8% vs. 3.1%, aOR 1.16 [0.81, 1.68]). Mixed results were detected for other pregnancy outcomes. Women with low leisure-time PA had excess risk of preterm birth (aOR 1.31 [1.05, 1.63]), but not higher rates of spontaneous preterm birth (OR 1.17 [0.88, 1.54]) or preeclampsia (OR 1.18 [0.91, 1.53]). Those with late decreasing leisure-time PA had higher risk of SGA (aOR 1.37 [1.05, 1.79]) compared to women with high activity.

**Table 4:** Crude and adjusted^a^ odds ratios for pregnancy outcomes among women 20+ weeks of gestation according to leisure-time physical activity trajectory class across nuMoM2b study visits

Pregnancy outcome	Pregnancy outcome	Crude odds ratios		Adjusted odds ratios	
Physical activity trajectory					
Class (Contrast)^b^	n/N (%)	Estimate (95% CI)	*p*-value	Estimate (95% CI)	*p*-value
Preterm birth (Nc = 9465, Na = 9278)					
High (referent)	247/3272 (7.5)	1.00	0.0175	1.00	0.1998
Early decreasing (versus referent)	90/980 (9.2)	1.24 (0.96–1.59)		1.12 (0.86–1.46)	
Early increasing (versus referent)	118/1300 (9.1)	1.22 (0.97–1.54)		1.09 (0.86–1.39)	
Late decreasing (versus referent)	194/2278 (8.5)	1.14 (0.94–1.39)		1.09 (0.89–1.33)	
Low (versus referent)	170/1635 (10.4)	1.42 (1.16–1.74)		1.31 (1.05–1.63)	
Spontaneous preterm birth (Nc = 9461, Na = 9274)					
High (referent)	160/3271 (4.9)	1.00	0.4563	1.00	0.5995
Early decreasing (versus referent)	54/978 (5.5)	1.14 (0.83–1.56)		1.09 (0.78–1.52)	
Early increasing (versus referent)	63/1300 (4.8)	0.99 (0.73–1.34)		0.95 (0.70–1.29)	
Late decreasing (versus referent)	105/2278 (4.6)	0.94 (0.73–1.21)		0.94 (0.72–1.21)	
Low (versus referent)	95/1634 (5.8)	1.20 (0.92–1.56)		1.17 (0.88–1.54)	
Preeclampsia (Nc = 9450, Na = 9263)					
High (referent)	163/3270 (5.0)	1.00	0.0098	1.00	0.6210
Early decreasing (versus referent)	64/976 (6.6)	1.34 (0.99–1.80)		1.05 (0.77–1.44)	
Early increasing (versus referent)	94/1299 (7.2)	1.49 (1.14–1.93)		1.21 (0.92–1.59)	
Late decreasing (versus referent)	149/2273 (6.6)	1.34 (1.06–1.68)		1.13 (0.89–1.43)	
Low (versus referent)	115/1632 (7.0)	1.44 (1.13–1.85)		1.18 (0.91–1.53)	
Preeclampsia or antepartum gHTN (Nc = 9450, Na = 9263)					
High (referent)	372/3270 (11.4)	1.00	0.0066	1.00	0.3719
Early decreasing (versus referent)	131/976 (13.4)	1.21 (0.98–1.49)		1.03 (0.82–1.29)	
Early increasing (versus referent)	186/1299 (14.3)	1.30 (1.08–1.57)		1.16 (0.95–1.41)	
Late decreasing (versus referent)	326/2273 (14.3)	1.30 (1.11–1.53)		1.14 (0.97–1.35)	
Low (versus referent)	227/1632 (13.9)	1.26 (1.05–1.50)		1.16 (0.96–1.40)	
GDM (Nc = 9314, Na = 9131)					
High (referent)	101/3245 (3.1)	1.00	0.0003	1.00	0.0001
Early decreasing (versus referent)	48/956 (5.0)	1.65 (1.16–2.34)		1.74 (1.21–2.52)	
Early increasing (versus referent)	48/1270 (3.8)	1.22 (0.86–1.73)		1.16 (0.81–1.68)	
Late decreasing (versus referent)	105/2226 (4.7)	1.54 (1.17–2.04)		1.50 (1.12–2.01)	
Low (versus referent)	92/1617 (5.7)	1.88 (1.41–2.51)		2.00 (1.47–2.73)	
SGA < 5th percentile (Nc = 9426, Na = 9239)					
High (referent)	123/3260 (3.8)	1.00	0.1649	1.00	0.2169
Early decreasing (versus referent)	45/974 (4.6)	1.24 (0.87–1.75)		1.13 (0.78–1.63)	
Early increasing (versus referent)	63/1294 (4.9)	1.31 (0.96–1.78)		1.15 (0.83–1.60)	
Late decreasing (versus referent)	116/2270 (5.1)	1.37 (1.06–1.78)		1.37 (1.05–1.79)	
Low (versus referent)	76/1628 (4.7)	1.25 (0.93–1.67)		1.08 (0.79–1.47)	

^a^Adjusted for age (linear and quadratic terms), race/ethnicity (white, non-Hispanic; black, non-Hispanic; Hispanic; Asian: and other), early pregnancy BMI (linear and quadratic terms), and smoking status 3 months prior to pregnancy. *P*-values are taken from logistic regression models

^b^Nc and Na present the number of observations used in calculating the crude odds ratios and the adjusted odds ratios, respectively

To account for the variability of being included in a group, models were additionally adjusted for the posterior probability of group assignment and results were unchanged. (Additional file 1: Table S1).

## Discussion

Our results from a large, multi-center, diverse and contemporary cohort of nulliparous women revealed that patterns of self-reported leisure-time PA change across pregnancy and that these patterns are related to risk of adverse pregnancy outcomes. Importantly, the third of women with the highest leisure-time PA levels throughout pregnancy had the lowest rates of complications. Almost 20% of women reported no leisure-time PA. These women were young, more likely to be of minority race/ethnicity, and more likely to have the highest rates of most complications. Gestational diabetes risk was associated with leisure-time PA patterns: women with high leisure-time PA and those who increased leisure-time PA in the first half of pregnancy had the lowest rates of GDM, independent of maternal characteristics.

Levels of activity prior to pregnancy are associated with patterns during pregnancy, although overall, women tend to decrease activity as gestation advances [3, 27]. Very little is known, however, regarding activity patterns across gestation and their relation to adverse pregnancy outcomes. Physical activity is a modifiable contributor to fitness, and fitness contributes to the integrity of the vascular and metabolic systems that undergo profound adaptation required for healthy pregnancy. Our results suggest that patterns of leisure-time PA at early, mid and late gestation may be associated with complications, particularly rates of GDM.

Consistent with other observational studies, few women in our contemporary cohort achieved recommended levels of leisure-time PA [28]. The patterns we detected, however, revealed that those with high leisure-time PA and those who increased their activity prior to mid-pregnancy had low rates of GDM. Our findings raise the possibility that women may be motivated to improve their health behaviors during pregnancy and that increasing levels of leisure-time PA could be associated with improved pregnancy health. Observational, intervention studies and systematic reviews consistently show that physical activity is associated with reduced risk of GDM [12, 29, 30]. Our study extends these to raise the possibility that increasing leisure-time PA prior to 24 weeks gestation (when GDM screening occurs) even at levels that fall short of recommendations, may be beneficial. This finding is consistent with evidence that increased activity is associated with lower glucose levels in women with GDM [12, 31]. Future work is needed to relate activity patterns to glucose, other biomarkers and weight gain to better understand mechanisms that may explain our findings.

Women with low leisure-time PA had high rates of all complications, including higher overall preterm birth, but not spontaneous preterm birth. This group did not have excess hypertensive disorders of pregnancy or small for gestational age births after accounting for maternal characteristics, the two leading reasons for medically indicated PTB. Taken together, these data suggest that other maternal or fetal health indications may contribute to the excess preterm birth we detected in women with low leisure-time PA. In addition, the excess risk of small for gestational age births detected only in women with late decreasing leisure-time PA may be due to reverse confounding, such that women may be under surveillance for impaired fetal growth and thus advised or choose to restrict their activity late in pregnancy. We were unable to disentangle these temporal indications for change.

Our results suggest that the pattern of leisure-time PA may be related to pregnancy health. Among the group with early increasing leisure-time PA, only 5% achieved recommended levels by 16 to 22 weeks’ gestation, and yet the pattern of increase was associated with lower rates of GDM compared to groups with sustained low or decreasing leisure-time PA. It is possible that health behaviors detected in a first pregnancy may persist post-partum and contribute to subsequent pregnancy health and long term maternal health. In addition, it is possible that maternal leisure-time PA patterns may be related to newborn health and adiposity risk [32, 33]. These long-term associations warrant investigation, and mothers in the current cohort are being followed to assess post-pregnancy maternal health and health behaviors [34].

Our observational study findings must be considered in light of limitations. Our activity data were self-reported on an instrument which was limited to only three structured activities and does not include more incidental, occupational, or light intensity activity. This approach may systematically underestimate activity levels in women that are more active. Evidence among non-pregnant adults who reported up to 4 activities indicates that collecting information on two leisure activities provides the most efficient balance for accuracy, and thus provides reassurance that the impact of this limitation may be small [35]. We also cannot rule out residual confounding or reverse causality, and cannot infer causality. We were unable to study factors that may be associated with changing leisure-time PA during pregnancy, such as provider advice to increase or decrease activity. For example, women at risk for spontaneous preterm birth (i.e. short cervix) may be advised to decrease activity [36, 37]. Our results did not indicate that women with low leisure-time PA had higher risk of spontaneous preterm birth, and thus the impact of this limitation may be modest. We also did not have information regarding pre-pregnancy leisure-time PA patterns which are likely informative. We also were unable to examine individual or community barriers such as occupational features and neighborhood safety that may deter activity during pregnancy. Strengths of our study include the large, diverse, contemporary cohort of nulliparous women followed prospectively; our ability to characterize patterns of leisure-time PA across pregnancy; and, validated adverse pregnancy outcomes derived from medical records.

## Conclusion

Our results indicate that women with sustained low leisure-time PA across pregnancy have excess risk of PTB and GDM compared to the women with leisure-time PA at higher levels across all trimesters of pregnancy. Of note, women that increased their leisure-time PA early in pregnancy had low rates of GDM similar to those in the high pattern group, raising the possibility that early pregnancy increases in leisure-time PA may be associated with improved pregnancy health.

## Additional file

Additional file 1: **Table S1.** Adjusted Odds Ratios for Adverse Pregnancy Outcomes Among Women 20+ Weeks of Gestation According to Physical Activity Trajectory Class Across nuMoM2b Study Visits. (DOCX 19 kb)

## Abbreviations

AIC: Akaike information criterion
aOR: Adjusted odds ratio
BIC: Bayesian information criterion
BMI: Body mass index
BRFSS: Behavioral Risk Factor Surveillance System
GDM: Gestational diabetes mellitus
GTT: Glucose tolerance testing
HDP: Hypertensive disorders of pregnancy
METs: Metabolic equivalents
OR: Odds ratio
PTB: Preterm birth
SGA: Small for gestational age
