# Supplementary material for: Patterns of leisure-time physical activity across pregnancy and adverse pregnancy outcomes
Source: Int J Behav Nutr Phys Act. 2018 Jul 11;15:68. doi: 10.1186/s12966-018-0701-5 (PMC6042402; doi:10.1186/s12966-018-0701-5)
Supplement: Supplementary file 1 — Table S1. Adjusted Odds Ratios for Adverse Pregnancy Outcomes Among Women 20+ Weeks of Gestation According to Physical Activity Trajectory Class Across nuMoM2b Study Visits. (DOCX 19 kb) [file 12966_2018_701_MOESM1_ESM.docx]

| Additional file 1: Table S1 | | | | | |
| --- | --- | --- | --- | --- | --- |
| Adjusted^1/^ Odds Ratios for Adverse Pregnancy Outcomes Among Women 20+ Weeks of Gestation  According to Physical Activity Trajectory Class Across nuMoM2b Study Visits | | | | | |
|  |  |  |  |  |  |
| Pregnancy Outcome  Physical Activity Trajectory   Class (Contrast)^2/^ | Pregnancy Outcome | Odds Ratios – Adjustment 1 | | Odds Ratios – Adjustment 2 | |
|  | n/N (%) | Estimate (95% CI) | p-value | Estimate (95% CI) | p-value |
|  |  |  |  |  |  |
| Preterm Birth (N1=9465, N2=9278) |  |  |  |  |  |
| High (referent) | 247/3272 (7.5) | 1.00 | 0.0085 | 1.00 | 0.1329 |
| Early decreasing (versus referent) | 90/980 (9.2) | 1.28 (0.99-1.66) |  | 1.16 (0.89-1.52) |  |
| Early increasing (versus referent) | 118/1300 (9.1) | 1.27 (1.00-1.60) |  | 1.13 (0.88-1.44) |  |
| Late decreasing (versus referent) | 194/2278 (8.5) | 1.12 (0.92-1.37) |  | 1.07 (0.88-1.31) |  |
| Low (versus referent) | 170/1635 (10.4) | 1.46 (1.18-1.79) |  | 1.35 (1.08-1.68) |  |
| Spontaneous Preterm Birth (N1=9461, N2=9274) | |  |  |  |  |
| High (referent) | 160/3271 (4.9) | 1.00 | 0.4157 | 1.00 | 0.5520 |
| Early decreasing (versus referent) | 54/978 (5.5) | 1.16 (0.84-1.60) |  | 1.11 (0.79-1.56) |  |
| Early increasing (versus referent) | 63/1300 (4.8) | 1.01 (0.74-1.37) |  | 0.97 (0.71-1.33) |  |
| Late decreasing (versus referent) | 105/2278 (4.6) | 0.93 (0.72-1.20) |  | 0.93 (0.72-1.21) |  |
| Low (versus referent) | 95/1634 (5.8) | 1.21 (0.93-1.58) |  | 1.19 (0.89-1.57) |  |
| Preeclampsia (N1=9450, N2=9263) |  |  |  |  |  |
| High (referent) | 163/3270 (5.0) | 1.00 | 0.0092 | 1.00 | 0.5872 |
| Early decreasing (versus referent) | 64/976 (6.6) | 1.36 (1.00-1.84) |  | 1.07 (0.78-1.47) |  |
| Early increasing (versus referent) | 94/1299 (7.2) | 1.51 (1.15-1.97) |  | 1.23 (0.93-1.63) |  |
| Late decreasing (versus referent) | 149/2273 (6.6) | 1.33 (1.05-1.67) |  | 1.12 (0.89-1.42) |  |
| Low (versus referent) | 115/1632 (7.0) | 1.46 (1.14-1.87) |  | 1.19 (0.91-1.55) |  |
| Preeclampsia or Antepartum gHTN (N1=9450, N2=9263) | | |  |  |  |
| High (referent) | 372/3270 (11.4) | 1.00 | 0.0080 | 1.00 | 0.3837 |
| Early decreasing (versus referent) | 131/976 (13.4) | 1.17 (0.94-1.45) |  | 1.00 (0.79-1.25) |  |
| Early increasing (versus referent) | 186/1299 (14.3) | 1.26 (1.04-1.53) |  | 1.12 (0.92-1.37) |  |
| Late decreasing (versus referent) | 326/2273 (14.3) | 1.32 (1.13-1.56) |  | 1.15 (0.98-1.36) |  |
| Low (versus referent) | 227/1632 (13.9) | 1.23 (1.03-1.47) |  | 1.13 (0.93-1.37) |  |
| GDM (N1=9314, N2=9131) |  |  |  |  |  |
| High (referent) | 101/3245 (3.1) | 1.00 | 0.0003 | 1.00 | 0.0002 |
| Early decreasing (versus referent) | 48/956 (5.0) | 1.62 (1.13-2.32) |  | 1.69 (1.16-2.47) |  |
| Early increasing (versus referent) | 48/1270 (3.8) | 1.20 (0.84-1.72) |  | 1.13 (0.78-1.65) |  |
| Late decreasing (versus referent) | 105/2226 (4.7) | 1.55 (1.17-2.06) |  | 1.52 (1.13-2.03) |  |
| Low (versus referent) | 92/1617 (5.7) | 1.86 (1.38-2.49) |  | 1.95 (1.42-2.69) |  |
| SGA<5th Percentile (N1=9426, N2=9239) | |  |  |  |  |
| High (referent) | 123/3260 (3.8) | 1.00 | 0.1642 | 1.00 | 0.1913 |
| Early decreasing (versus referent) | 45/974 (4.6) | 1.24 (0.87-1.77) |  | 1.11 (0.76-1.61) |  |
| Early increasing (versus referent) | 63/1294 (4.9) | 1.31 (0.96-1.80) |  | 1.13 (0.81-1.58) |  |
| Late decreasing (versus referent) | 116/2270 (5.1) | 1.37 (1.05-1.78) |  | 1.39 (1.06-1.81) |  |
| Low (versus referent) | 76/1628 (4.7) | 1.25 (0.93-1.69) |  | 1.06 (0.77-1.46) |  |
|  |  |  |  |  |  |
|  |  |  |  |  |  |
| 1/ The first adjustment includes the probability of class membership from the growth mixture model only. The second adjustment includes this, plus age (linear and quadratic terms), race/ethnicity (white, non-Hispanic; black, non-Hispanic; Hispanic; Asian: and other), early pregnancy BMI (linear and quadratic terms), and smoking status 3 months prior to pregnancy. P-values are taken from logistic regression models. | | | | | |
| ^2/^ N1 and N2 present the number of observations used in calculating the adjusted odds ratios, 1 and 2, respectively. | | | | | |
